# Irradiance-dependent UVB Photocarcinogenesis

**DOI:** 10.1038/srep37403

**Published:** 2016-11-21

**Authors:** Cheng-Che E. Lan, Ching-Shuang Wu, Shu-Mei Huang, Chin-Han Wu, Hsiao-Chi Lai, Yu-Ting Peng, Pao-Sheng Hou, Hui-Jun Yang, Gwo-Shing Chen

**Affiliations:** 1 Department of Dermatology, Kaohsiung Medical University Hospital, Department of Dermatology, College of Medicine, Kaohsiung Medical University, Kaohsiung, Taiwan; 2 Department of Dermatology, Kaohsiung Municipal Ta-Tung Hospital, Kaohsiung Medical University, Kaohsiung, Taiwan; 3 Lipid Science and Aging Research Center, Kaohsiung Medical University, Kaohsiung, Taiwan; 4 Center of Environmental Medicine, Kaohsiung Medical University, Kaohsiung, Taiwan; 5 Department of Medical Laboratory Science and Biotechnology, Kaohsiung Medical University, Kaohsiung, Taiwan

## Abstract

Ultraviolet B (UVB) radiation from the sun may lead to photocarcinogenesis of the skin. Sunscreens were used to protect the skin by reducing UVB irradiance, but sunscreen use did not reduce sunburn episodes. It was shown that UVB-induced erythema depends on surface exposure but not irradiance of UVB. We previously showed that irradiance plays a critical role in UVB-induced cell differentiation. This study investigated the impact of irradiance on UVB-induced photocarcinogenesis. For hairless mice receiving equivalent exposure of UVB radiation, the low irradiance (LI) UVB treated mice showed more rapid tumor development, larger tumor burden, and more keratinocytes harboring mutant p53 in the epidermis as compared to their high irradiance (HI) UVB treated counterpart. Mechanistically, using cell models, we demonstrated that LI UVB radiation allowed more keratinocytes harboring DNA damages to enter cell cycle via ERK-related signaling as compared to its HI UVB counterpart. These results indicated that at equivalent exposure, UVB radiation at LI has higher photocarcinogenic potential as compared to its HI counterpart. Since erythema is the observed sunburn at moderate doses and use of sunscreen was not found to associate with reduced sunburn episodes, the biological significance of sunburn with or without sunscreen use warrants further investigation.

Exposure to sun radiation is the most important environmental risk factor for inducing skin cancers^1^,^2^. Among the different regions of solar radiation, ultraviolet (UV) B radiation is most closely associated with photocarcinogenesis. DNA is a natural chromophore of UVB radiation. It was demonstrated that UVB induces DNA damage by forming photoproducts that if unrepaired, leads to mutations in the epidermis and results in development of skin cancers^3^. Additionally, UVB radiation stimulates inflammatory erythema on the skin that contributes to development of sunburn. While sunburn may not be part of the mechanism of cancer, the history of sunburn has close association with development of skin cancers^4^. Sunscreens were introduced to protect the skin from UV radiation by extending the duration required to induce sunburn. There are different types of sunscreen, designed to either absorb or deflect a proportion of incoming UV photon energy. An intriguing phenomenon frequently reported is that the use of sunscreen has not been associated with reduced sunburns. More specifically, it was found that although the prevalence of sunscreen use increased from 1997 to 2007, this increase was not accompanied by reduction in sunburn episodes^5^. Sunscreen use has been associated with longer sun exposure especially for intentional sun seekers and therefore provided a possible link between frequent sunscreen use and more sunburns^6^,^7^.

Although clinical trials have shown that sunscreen use is associated with reduction of skin cancer development, particularly squamous cell tumors^8^,^9^, an increasing trend of skin cancer, including squamous cell carcinoma, has been observed in the real world setting^10^,^11^,^12^, despite the increasing prevalence of sunscreen use. Frequently, suboptimal behavioral pattern of sunscreen use, including not putting on enough or failure to reapply, is proposed to contribute to sunscreen failure^13^,^14^. However, it is difficult to accurately document behavioral patterns in clinical studies. Since sunscreen use is the most commonly employed sun protective behavior^15^, it is important to determine if sunburn episodes with sunscreen use produce similar damages to the skin as compared to sunburn episodes without sunscreen use. As aforementioned, sunscreens, either chemically or physically, reduce the UVB photons from penetrating the skin. This scenario is similar to reduced irradiance (photon density; mW/cm^2^) from a UVB radiation source. Intriguingly, we had recently demonstrated that UVB radiation at equivalent surface exposure (mJ/cm^2^) but different irradiance imparts different biologic effects on immature pigment cells^16^. Erythema is the observed sunburn at moderate doses. It has demonstrated that UVB-induced erythema depends on surface exposure delivered regardless of irradiance^17^,^18^,^19^. This phenomenon allowed us to examine the damaging effects of UVB radiation (same surface exposure) on the skin with different irradiance in a laboratory setting using a neutral density physical filter (a filter that does not change the spectrum) that avoids aforementioned potential confounders in animal studies using sunscreen to reduce irradiance. We hypothesized that equivalent UVB surface exposure delivered at different irradiance may have significant impact on its photocarcinogenic potential. The current study was launched to address this important health issue.

## Results

### Hairless mice receiving low irradiance (LI) UVB treatment are more susceptible to develop skin tumors as compared to their high irradiance (HI) UVB treated counterparts

**Figure 1:**
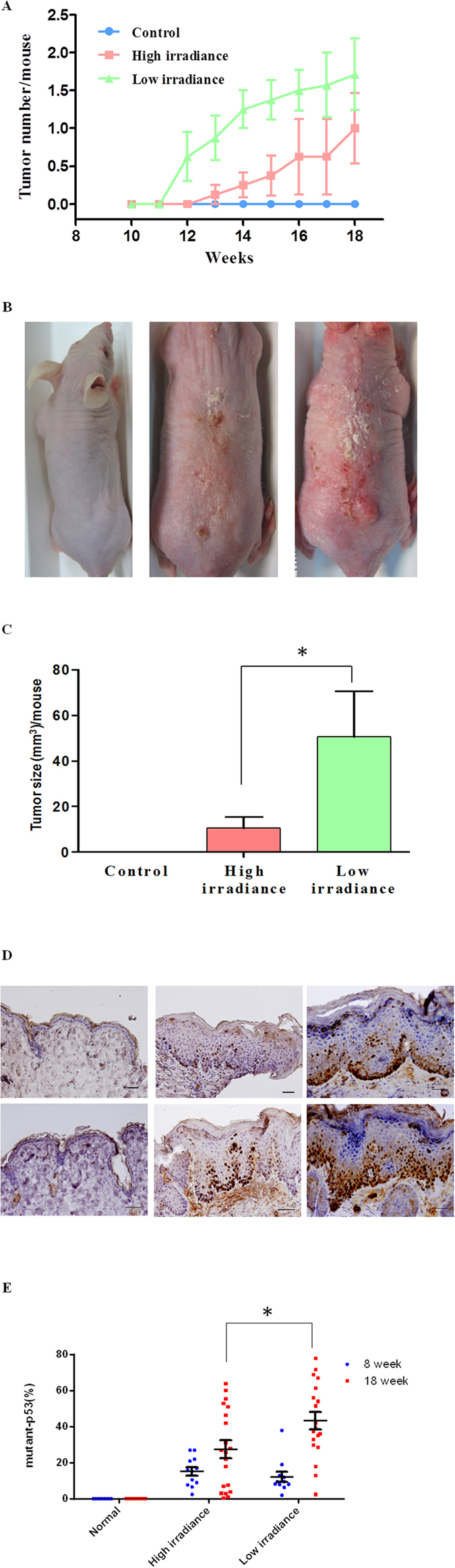
Effect of irradiance on UVB-induced skin tumor. The hairless mice were treated with high or LI UVB radiation at equivalent surface exposure (200 mJ/cm^2^) three times per week. (**A**) The number of skin tumors per mouse after indicated UVB treatment. (**B**) The representative presentation of dorsal mouse skin after 18 weeks of indicated UVB treatment. Left: control mouse; middle: high irradiance (HI) UVB treated mouse; right: low irradiance (LI) UVB treated mouse. (**C**) The tumor burden per mouse after 18 weeks of indicated UVB treatment. (**D**) The expression of mutant p53 in the normal-appearing skin of hairless mice after indicated UVB treatment. Representative presentation of immunohistochemical staining of p53 (PAb240 antibody) on dorsal skin of control (left), HI UVB treated (middle), and LI UVB (right) mice after 8 wks (top panel) and 18 wks (bottom panel) of indicated UVB treatment. (**E**) The quantitative analyses of mutant p53 expression on the dorsal skin of control, HI, and LI UVB treated mice after 8 wks and 18 wks of indicated UVB treatment. Scale bar indicates 100 μm. *Indicates p < 0.05.

To examine if equivalent exposure of UVB radiation delivered at different irradiance has significant impact on its photocarcinogenic potential, a hairless mouse model was used. All the mice treated with LI UVB developed tumor after 14 wks (n = 7) (Fig. 1A). On the other hand, it took 18 wks for more than half of the mice receiving HI UVB treatment to developed skin tumor (n = 7). At the end of 18 wks, the LI UVB treated group showed larger tumor burden per mouse (more than 4 folds) as compared to their HI UVB treated counterpart (Fig. 1B and C). UVB irradiation was stopped after 18 wks since most of the mice have developed skin tumor, and in clinical settings, skin tumors were usually treated in a timely manner after detection.

### The normal appearing skin of the LI UVB treated mice showed significantly higher number of keratinocytes harboring mutant p53 as compared to their HI UVB treated counterparts

Previous studies demonstrated that inactivation or mutation of the p53 tumor suppressor gene plays a critical role in the development of human and mouse UV-induced skin cancers. Mutations observed at dipyrimidine sites, especially C -> T or CC -> TT transitions, are caused by UV radiation and termed “UV signature” mutations^20^. In this study, Pab240 monoclonal antibody was used to detect UVB-induced p53 mutation. The Pab240 antibody recognizes an epitope that is exposed in mutant p53 proteins due to a conformational exchange, while the epitope is cryptic in wild type p53 protein^21^. Normal appearing skin specimens from hairless mice were obtained at 8 wks and 18 wks after initiation of UVB treatment. Mutant p53 expression in keratinocytes was found scattered at the basal layer of the normal appearing mouse skin after 8 wks of UVB treatment. No significant difference was found between the HI and LI UVB treated groups (Fig. 1D and E). On the other hand, the LI UVB treated mice showed significantly (p < 0.05) higher number of keratinocytes harboring mutant p53 in the normal appearing skin as compared to their HI treated counterpart 18 wks after UVB irradiation (Fig. 1D and E). These results suggest that at same surface exposure, LI UVB treatment is more likely to allow survival and proliferation of keratinocytes harboring mutant p53 as compared to its HI UVB counterpart.

### Cultured keratinocytes demonstrated more DNA damage after LI UVB radiation as compared to their HI UVB radiated counterpart after equivalent surface exposure

**Figure 2:**
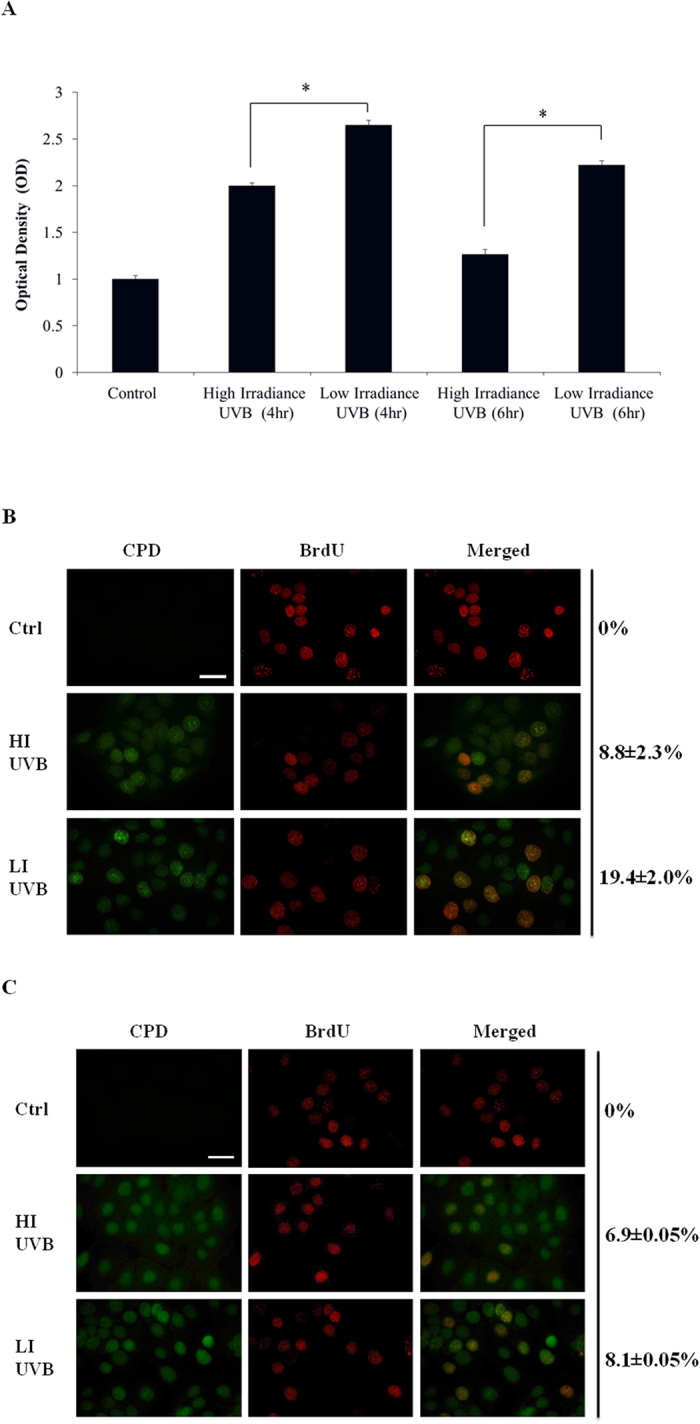
CPD and Brdu expression in cultured keratinocytes after indicated UVB irradiation. (**A**) The CPD lesions of normal keratinocytes at 4 and 6 hr after high irradiance (HI) and low irradiance (LI) UVB (23 mJ/cm^2^) treatment as determined by ELISA specific for CPD. (**B**) Double labeling of HaCaT keratinocytes 3 hr after HI and LI UVB (23 mJ/cm^2^) treatment with anti-CPD antibody (green) and anti-BrdU (red). (**C**) Double labeling of ERK inhibitor (U0126; 5 μM) pretreated HaCaT keratinocytes 3 hr after HI and LI UVB (23 mJ/cm^2^) treatment with anti-CPD antibody (green) and anti-BrdU (red). The numbers show percentage (mean ± SD) of BrdU + CPD keratinocytes.

To elucidate the biological differences between HI and LI UVB radiation in the context of photocarcinogenesis, *in vitro* cell models were used. UVB radiation results in DNA damages that interfere with the critical cellular processes including transcription and replication, leading to disrupted cell cycle progression. More importantly, replications of cells with DNA damages ultimately lead to formation of skin cancers. Cyclopyrimidine dimer (CPD) formation is the hallmark of DNA damage after UVB radiation and played a major role in photocarinogenesis^22^. Previously, we had shown that LI UVB radiation induces more CPD formation as compared to its HI counterpart if same surface exposure was administered^16^. Using cultured normal keratinocytes, it was found that at both 4 and 6 hrs after UVB radiation, the LI UVB treated group resulted in significantly higher CPD levels as compared to their HI UVB treated counterpart (Fig. 2A). It should be noted that similar findings were obtain from cultured HaCaT keratinocytes, a model of the precancerous skin lesion harboring mutated p53^23^. More specifically, the CPD level of LI UVB treated HaCaT keratinocytes at 4 hr after UVB radiation was 1.37 ± 0.01 folds of their HI UVB treated counterpart (p < 0.05). These results suggest that at equivalent surface exposure, LI UVB induced more CPD formation in keratinocytes as compared its HI UVB counterpart. Alternatively, impaired CPD repair after LI UVB irradiation as compared to its HI UVB counterpart may also contribute to this phenomenon. Since our previous study already demonstrated more CPD formation immediately after LI UVB irradiation as compared to its HI UVB counterpart after equivalent exposure^16^, the potential impact of irradiance on DNA repair requires further investigation. It should be noted that in animal studies, after 8 weeks of regular UVB treatment, the LI UVB exposed mice skin showed higher portion of epidermal keratinocytes harboring CPD as compared to their HI UVB treated counterpart (Supplementary file Fig. S3) although no statistical significance was found (p = 0.056). As CPD repair may depend on the timing of biopsy, multiple biopsies performed at different time points after UVB exposure may be necessary to document the statistical difference between HI and LI UVB irradiated mice skin in terms of CPD levels.

### LI UVB radiation allowed more HaCaT keratinocytes to survive than their HI UVB treated counterpart despite inducing more DNA damages

Since UVB induced DNA damages in both normal and HaCaT keratinocytes, we next determined the keratinocyte cell number after indicated UVB treatment. The cell number of cultured normal keratinocytes after HI and LI UVB (26 mJ/cm^2^) radiation were 115% ± 1.6% and 111% ± 3.6 of control, respectively, with no significant difference between the two groups. On the other hand, the cell number of HaCaT keratinocytes showed 82.2% ± 1.9% and 89.8% ± 2.2% of control after HI and LI UVB radiation, respectively. The LI UVB treated HaCaT keratinocytes demonstrated significantly higher cell survival as compared to their HI UVB treated counterpart (p < 0.05). This result is counterintuitive as LI UVB radiation induced significantly higher CPD formation in HaCaT keratinocytes as compared to its HI UVB counterpart. We next determined the apoptotic events induced by indicated UVB radiation. No significant difference in apoptosis was found among cultured normal keratinocytes after UVB treatment regardless of irradiance (data not shown). On the other hand, HaCaT keratinocytes showed increased cellular apoptosis after indicated UVB radiation with LI UVB induced significantly more apoptotic event (p < 0.05) as compared to its HI counterpart. More specifically, the LI and HI UVB treatment (26 mJ/cm^2^) induced 5.48 ± 0.01% and 3.40 ± 0.07% cells to undergo apoptosis, respectively. These results indicated that normal keratinocytes are more resistant to UVB induced damages as compared to HaCaT keratinocytes. Similar findings were previously reported by Faurschou *et al*.^24^. The differences in genetic background between primary normal and HaCaT keratinocytes likely contributed to this phenomenon. However, it is intriguing to note that while LI UVB radiation induced significantly higher CPD formation and more apoptotic events in HaCaT keratinocytes, the HaCaT cell number after LI UVB treatment was significantly higher than their HI UVB treated counterpart. Since the differences in keratinocytes harboring mutant p53 were noted between the HI and LI UVB treated mice after 18 wk of UVB radiation, we focused our subsequent studies using HaCaT keratinocytes to present premalignant cell harboring dysfunctional p53^23^.

### LI UVB treated HaCaT keratinocytes showed more CPD and BrdU co-expression as compared to their HI UVB treated counterpart

To determine if LI UVB radiation allowed more HaCaT keratinocytes harboring CPD to enter cell cycle, immunocytochemical staining was performed. Similar to previous results (Fig. 2A), significantly more (p < 0.05) LI UVB treated HaCaT keratinocytes contained CPD (25.8 ± 6.5%) as compared to their HI UVB treated counterpart (15.5 ± 3.5%). For BrdU staining, UVB radiation significantly reduced (p < 0.05) BrdU staining in HaCaT keratinocytes. More specifically, higher portion of LI UVB treated HaCaT keratinocytes incorporated BrdU (22.1 ± 1.2%) as compared to their HI UVB treated counterpart (15.3 ± 3.5%). The percentage of HaCaT keratinocytes showing co-expression of CPD and BrdU was significantly higher (p < 0.05) in LI UVB treated group as compared to their HI UVB treated counterpart (Fig. 2B). This result suggested that LI UVB treatment renders HaCaT keratinocytes harboring unrepaired DNA to enter cell cycle as compared to their HI UVB treated counterpart when same surface exposure was administered.

### LI UVB treated HaCaT keratinocytes showed cell cycle progression with reduced G2M phase as compared to their HI UVB treated counterpart via enhanced extracellular signal-regulated kinases (ERK) signaling

**Figure 3:**
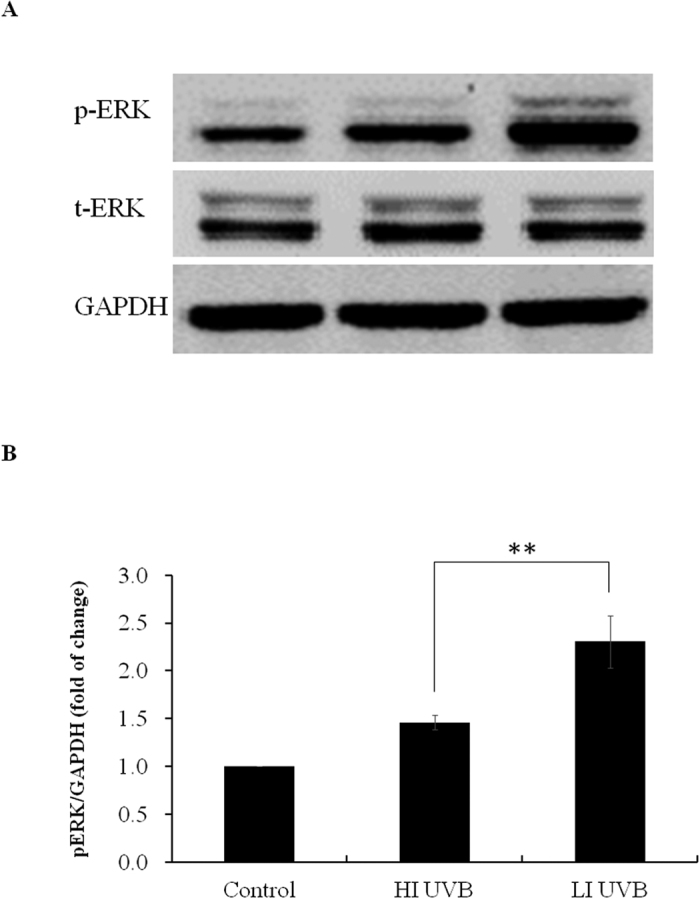
The expression of ERK pathway after indicated UVB treatment. (**A**) Cultured HaCaT keratinocytes were treated with high irradiance (HI) and low irradiance (LI) UVB (23 mJ/cm^2^) treatment. The protein extracts were analyzed with western blotting using antibodies against phosphorylated ERK (p-ERK), total ERK (t-ERK), and GAPDH. (**B**) Densitometric analyses of (**A**) n = 3. **Indicates p < 0.05.

As aforementioned, UVB radiation may result in disrupted cell cycle progression. Therefore, we next evaluated the cell cycle distribution of HaCaT keratinocytes after indicated UVB radiation. As demonstrated in Table 1, after LI UVB radiation, the HaCaT keratinocytes showed enhanced S phase and reduced G2M phase distribution as compared to their HI UVB treated counterpart. In parallel, the BrdU incorporation of HaCaT keratinocytes was significantly higher (p < 0.05) in the LI UVB treated group as compared to their HI UVB treated counterpart. More specifically, the BrdU incorporation of LI UVB treated HaCaT keratinocytes was 114.5% ± 4.7% of their HI UVB treated counterpart. Slower progression through S phase of cells with damaged DNA may have contributed to these phenomena. Previously, it has been shown that ERK signaling plays an important role in cutaneous SCC survival and growth^25^,^26^,^27^. Therefore, we next evaluated the expression of ERK signaling in our experimental conditions. UVB radiation significantly increased the phosphorylated-ERK (pERK) expression in HaCaT keratinocytes. Moreover, at equivalent surface exposure, the increase in pERK was significantly higher in LI UVB treated HaCaT keratinocytes as compared to their HI UVB treated counterpart while no significant difference was found between the total ERK expression (Fig. 3A and B). To validate the functional role of ERK signaling in our study, HaCaT keratinocytes were pretreated with ERK inhibitor U0126 before indicated UVB treatment. Pretreatment with U0126 reduced the HaCaT cell number after UVB radiation. More specifically, the cell number of HaCaT keratinocytes was 66.1% ± 1.3% and 63.8% ± 1.4% of control after HI and LI UVB radiation, respectively, with no significant difference between these groups. It is clear that LI UVB treated HaCaT keratinocytes no longer have higher cell survival as compared to their HI UVB treated counterpart after ERK inhibitor pretreatment. Using immunocytochemical staining, it was demonstrated that pretreatment with ERK inhibitor significantly reduced BrdU expression in both LI and HI UVB treated groups. Additionally, no significant difference was found between the percentages of HaCaT keratinocytes demonstrating co-expression of CPD and BrdU after LI and HI UVB exposures. (Fig. 2C) Corroborating with these results, the cell cycle distribution of LI UVB irradiated HaCaT keratinocytes pretreated with ERK inhibitor no longer demonstrated increased S phase distribution as compared to their HI UVB treated counterpart (Table 2). In parallel, after pretreatment with ERK inhibitor, the BrdU incorporation of LI UVB treated HaCaT keratinocytes was 91.4 % ± 2.4 % of their HI UVB treated counterpart, with no significant difference between the two groups. These results indicated that LI UVB radiation enhanced HaCaT keratinocyte survival via ERK cascade, despite inducing more cell damages.

### HI UVB treatment induced more immune suppression in mice as compared to its LI UVB counterpart

**Figure 4:**
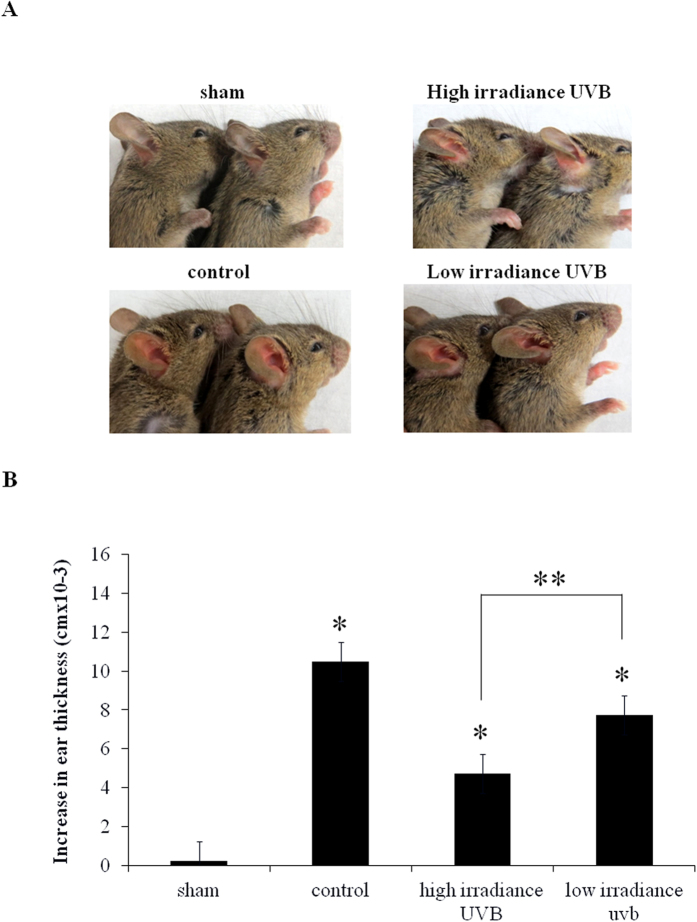
Contact hypersensitivity (CHS) was performed to compare the levels of immunosuppression after indicated UVB treatment. (**A**) The representative presentation of ear swelling response after indicated treatment. The increase in ear thickness was defined as the amount of swelling of the 2,4,-dinitrofluorobenzene (DNFB)-challenged ear as compared to the thickness of vehicle treated ear. Upper Left: control mouse with no prior sensitization; Lower Left: Positive control mouse with prior sensitization; Upper Right: high irradiance (HI) UVB treated mouse before sensitization; Lower Right: low irradiance (LI) UVB treated mouse before sensitization. (**B**) The quantitative analyses of increase in ear thickness after indicated treatment. Sham: control mice with no prior sensitization; Control: mice with prior sensitization; HI UVB: mice treated with HI UVB prior to sensitization; LI UVB: mice treated with LI UVB treated mice prior to sensitization. *Indicates p < 0.05 as compared to sham treated group; **Indicates p < 0.05.

It has been recognized that both cell damage and immune suppression played important roles in the photocarcinogenic process of the skin^28^. In this study, we demonstrated that at equivalent surface exposure, LI UVB radiation has more photocarcinogenic potential as compared to its HI UVB counterpart. Since we demonstrated that LI UVB radiation induced more cell damage but enhanced survival signal that allows DNA damaged cells to enter cell cycle and survive as compared to HI UVB, we next determined if immune suppression also contributed to the enhanced carcinogenic capacity of LI UVB radiation. UVB-induced systemic immune suppression was evaluated by contact hypersensitivity model. As demonstrated in Fig. 4, HI UVB treated mice demonstrated significantly greater immune suppression as compared to its LI UVB treated counterpart after equivalent surface exposure. More specifically, the increase in ear thickness is significantly lower in the HI UVB treated mice as compared to their LI UVB treated counterpart. Therefore, although LI UVB radiation has more carcinogenic potential on the skin as compared to its HI UVB counterpart when same surface exposure was administered, the immune suppressive effect did not play a significant role contributing to this phenomenon.

## Discussion

In this study, we demonstrated that at equivalent surface exposure, UVB radiation administered at LI is more likely to induce skin carcinogenesis as compared to its HI counterpart using the hairless mouse model. This phenomenon deviates from the conventional reciprocity law in which one assumes the effect of radiation will be proportional to the total dose delivered, where dose (mJ/cm^2^) is the product of irradiance (mW/cm^2^) times exposure duration (seconds)^29^. Similar observations describing “reciprocity failure” have been reported previously without mechanistic investigation^29^,^30^. Both DNA damage and immune suppression are key factors that lead to UV-induced carcinogenesis. In this study, we demonstrated that at equivalent surface exposure, HI UVB radiation induced more immune suppressive effect in mice as compared to their LI UVB treated counterpart. These results corroborated with previous report by Novak *et al*.^31^ using *in vitro* model evaluating UVB-induced T-Cell apoptosis, and very recently Iida *et al*.^17^ also reported similar finding using C3H/HeNJcl mice. Therefore, immune suppression played a limited role leading to greater photocarcinogenic capacity induced by LI UVB radiation as compare to its HI UVB counterpart at equivalent surface exposure. On the other hand, the hairless mice treated with LI UVB demonstrated higher expression of mutant p53 protein in the normal appearing skin as compared to their HI UVB treated counterpart. It has previously been shown that there is a causal relationship between the amount of mutant p53 expressing keratinocyte patches and the development of skin carcinoma^32^. Therefore, our results strongly suggest that LI UVB radiation is more likely to allow survival and proliferation of keratinocytes harboring UVB-induced DNA damage as compared to its HI counterpart when equivalent surface exposure is delivered. To elucidate the mechanisms involved in this process, cultured keratinocyte models were used. In this study, it was shown that at certain time points after equivalent surface exposure, LI UVB radiation results in higher CPD levels in keratinocytes as compare to their HI UVB treated counterpart, and enhanced ERK signaling promoted survival of keratinocytes harboring unrepaired CPD to enter cell cycle and survive. These results provided a reasonable rationale explaining the increased proportion of keratinocytes harboring mutant p53 in the epidermis of normal appearing skin of mice receiving LI UVB radiation as compared to their HI UVB treated counterpart. Collaborating with our result, it was recently demonstrated that at equivalent exposure dose, single LI UVB treatment induced higher levels of 8-hydroxy-2′deoxy-guanosine expression, a recognized biomarker for DNA damage and oxidative stress, in the epidermis of hairless mice as compared to their HI UVB treated counterpart^17^. Taken together, these events contributed to enhanced UVB-induced photocarcinogenesis resulting from LI UVB radiation as compared to its HI counterpart when equivalent surface exposure was administered. It is known that UVB radiation at equivalent exposure dose but different irradiance may have different impacts on aryl hydrocarbon receptor (AhR) signaling and DNA damage^16^. Both aforementioned events may serve as the initiating signal after UVB radiation. Exogenous TCDD, a potent AhR activator, failed to significantly alter the pERK expression in cultured HaCaT keratinocytes. (data not shown) Previously, UV-induced DNA damage, particularly CPD formation, has been shown to trigger cytokine production^33^, alter proteinase expression^34^, and served as initiating molecular signaling within the cells^35^. Therefore, increased DNA damage induced by LI UVB radiation may contribute to its increased carcinogenic potential as compared to its HI counterpart at equivalent exposure dose. Other factors, including differences in levels of oxidative stress and direct membrane receptors activation may also participate in this process. Further studies are warranted to clarify these issues.

The results derived from this study may have important clinical implications. First of all, from epidemiology studies, it was demonstrated that although the prevalence of sunscreen use is increasing, the incidence of sunburn has not decreased. In this study, it was demonstrated that at equivalent surface exposure, UVB radiation at LI has higher photocarcinogenic potential as compared to its HI counterpart. Since erythema is the observed sunburn as moderate doses and use of sunscreen was not found to associated with reduced sunburn episodes, the biological significance of sunburn with or without sunscreen use warrants further investigation.

## Methods

### Photocarcinogenesis animal model

Six- to 8-week-old hairless SKH-hr1 mice were purchased from Charles River Laboratories Inc. (Wilmington, MA, USA). The UVB-mediated biological effects were evaluated by specific UVB surface exposure (mJ/cm^2^) as determined by multiplication of UV irradiance (mW/cm^2^; measured by UVB meter (National Biological Corporation, Twinsburg, OH)) and UV exposure time (seconds). The treatments for hairless mice included: 1) sham irradiation; 2) high irradiance (HI) UVB 200 mJ/cm^2^ irradiation; 3) low irradiance (LI) (with 50% irradiance reduction after application of neutral density physical irradiance filter) UVB 200 mJ/cm^2^ irradiation. For UVB irradiation, the mice were irradiated with UVB on the dorsal skin three times a week for 18 weeks. The UVB irradiation was performed using MEL308 (DEKA, Firenze, Italy; with wavelengths spectrum shown in Supplementary file Fig. S1). For irradiation of the mice skin, the irradiation handpiece was placed directly on top of the back of the mice. Since the irradiation field of the handpiece was 30 cm^2^, the entire back of the mouse was able to receive irradiation during the treatment. For LI UVB radiation, the physical filter capable of 50% irradiance reduction with holders on the sides to secure its location (custom-manufactured by National Applied Research Laboratories Instrument Technology Research Center, National Science Council, Taiwan) was placed directly in front of irradiation handpiece (the wavelengths spectrum and irradiance before and after filter placement were shown in Supplementary file Fig. S2). The reduction of irradiance from UVB radiation was validated using the UVB meter (National Biological Corporation, Twinsburg, OH) prior to experimentations. The animal studies were approved by the ethic committee of Kaohsiung Medical University.

### Immunohistochemical staining

The mouse epidermis was obtained at indicated time for immunohistochemical staining. Four-micron paraffin sections were deparaffinized in xylene and rehydrated in graded alcohol dilutions. Endogenous peroxidase activity was blocked by incubation with 3% H_2_O_2_ for 5 min. Antigen retrieval was performed by pressure cooking for 10 min (121°C, 1.2 kg/cm^2^) in Tris-EDTA buffer (pH9.0, with 0.1% Tween-20). The slides were then incubated with mouse anti-p53 (Pab 240) antibody (1:200 dilution; Santa Cruz Biotechnology, Santa Cruz, Calif) at 4 °C overnight. Antibody reactions were detected with an HRP-linked polymer Envision detection system (DAKO, Glostrup, Denmark) followed by hematoxylin counterstaining. Ten high-power fields for each section were randomly selected for analyses and the positive-staining keratinocytes were determined under the microscope (Olympus DP70, Olympus Optical Co., Ltd., Tokyo, Japan). The calculating formulation was as follows: the expression of mutant p53 expression in the epidermal keratinocyte/total epidermal keratinocyte x 100%.

### Contact hypersensitivity model (CHS)

Each experimental group consisted of five C3H/HeN mice. Three sets of experiments were carried out independently. The shaved back of mice was irradiated with UVB 150 mJ/cm^2^ for 4 consecutive days. Twenty four hours after the last UVB irradiation, the mice were sensitized by painting 50 μl DNFB (0.5% in acetone/olive oil, 4/1) on the shaved back. Five days later, ear challenge was performed by applying 20 μl of 0.3% DNFB to the right ear and an acetone/olive oil vehicle was applied to the left ear. Ear swelling was measured with a spring-loaded micrometer (Mitsutoyo, Kawasaki, Kanagawa, Japan) 24 h after the challenge. The ear-swelling response was measured in a blinded manner. Sensitized and challenged mice served as the positive control group, whereas the challenge only mice (without prior sensitization) served as negative control. CHS was defined as the amount of swelling of the DNFB-challenged ear compared with the thickness of the vehicle-treated ear in animals and the result was expressed as centimeters × 10^−3^ (mean ± SD)^36^,^37^.

The study was approved by the ethics committee of Kaohsiung Medical University and conducted according to the Declaration of Helsinki.

### Culture of normal and HaCaT keratinocytes

Normal human keratinocytes were obtained from healthy adult foreskin and cultured as previously described^25^. The study was approved by the ethics committee of Kaohsiung Medical University Hospital and conducted according to the Declaration of Helsinki. Written informed consent was obtained from all patients prior to participation in this study. HaCaT keratinocytes were grown in Dulbecco's Modified Eagle Medium (DMEM, Gibco) supplemented with 10 % FBS. Cultured cells were maintained in a humidified incubator with 5% CO_2_ and atmosphere at 37°C, and the medium was changed every 2 days.

### Treatment and experiments of cultured keratinocytes

During UVB irradiation, the lids of the culture dishes or plates were removed and the culture medium was replaced with phosphate buffered saline (PBS) to avoid formation of medium derived toxic photoproducts induced by UV exposure. The irradiation handpiece was placed directly on top of culture apparatus during UVB treatment. For LI UVB irradiation, the physical filter was placed as described above, and reduction of irradiance was confirmed with UVB meter (National Biological Corporation, Twinsburg, OH). For ERK signaling inhibition, cultured HaCaT keratinocytes were pretreated with 5 μM U0126 (Calbiochem; EMD Chemicals, San Diego) for 2 h before UVB irradiation.

### Cyclobutane pyrimidine dimmer (CPD) formation

At indicated time after UVB irradiation, the cultured keratinocytes were harvested and genomic DNA extracted by QIAamp DNA blood extraction kit (Qiagen, GmbH, Hilden, Germany) according to the manufacturer’s protocol. Extracted DNA samples were performed using the high sensitivity CPD ELISA kit for CPD quantification (Cosmo Bio Co., LTD, Tokyo, Japan).

### Cell number

Twenty-four h after treatments, cultured keratinocytes were fixed and stained with 100 μl crystal violet solution (0.5% crystal violet, 0.85% NaCl, 50% ethanol in 5% PBS) for 15 min. After washing with PBS, the dye was extracted with 33% acetic acid, and absorbance was measured at 595 nm (Model 450; Bio-Rad, Hercules, CA).

### Detection of cellular apoptosis

A commercially available kit (FITC Annexin V Apoptosis Detection Kit; BD Pharmingen™, NJ) was used to determine cell apoptosis according to the manufacturer’s instructions. The results were analyzed by flow cytometry (EPICS®XL-MCL™; Beckman Coulter) using FlowJo software (Treestar, Inc., San Carlos, CA).

### BrdU incorporation assay

A commercially available kit (Cell Proliferation ELISA, BrdU (colorimetric); Roche, Basel, Schewiz) was used to determine BrdU incorporation according to the manufacturer’s instructions.

### Immunocytochemical staining of HaCaT keratinocytes

Cultured HaCaT keratinocytes were seeded onto the coverslip (4 × 10^6^ cells/ml). Two and half hours after indicated treatment, BrdU solution (10 nM; Sigma) were added to HaCaT keratinocytes for incorporation at 37°C. After 30 min, the HaCaT keratinocytes were fixed with 4% (v/v) paraformaldehyde for 20 min at room temperature. Cultured HaCaT keratinocytes were then washed three times with PBS and treated with 0.1% Triton X-100 and 0.05% Tween20 for 30 min at room temperature. After three washings with PBS, the HaCaT keratinocytes were blocked with 1% BSA for 30 min at room temperature. Subsequently, the cells were incubated with CPD antibody (1:1000; Cosmo Bio Co., Ltd, Tokyo, JP) overnight at 4°C and conjugated with Alexa Fluor 488 goat anti-mouse IgG (1:1000; Life Technologies, Rockville, MD, USA) for 30 min at room temperature. 2N HCl were used to denature cellular DNA for 30 min at room temperature. The cells were blocked with 1% BSA again and incubated with anti-BrdU antibody (1:1000; Abcam, Cambridge, MA) for 1 h at room temperature and conjugated with Alexa Fluor 594 goat anti-rat IgG (1:1000; Invitrogen, Carlsbad, CA) for 30 min at room temperature. 100 ng/ml of DAPI (Invitrogen) were then added and incubated for 10 min at room temperature. All fluorescencent images were obtained using Olympus DP70 fluorescence microscope (Olympus Optical. Co., Ltd., Tokyo, Japan). For quantification of CPD-, BrdU-, and BrdU/CPD-retaining cells, ten randomly selected views were examined. The occurrences of CPD-, BrdU, and BrdU/CPD-retaining cells were expressed as a percentage of total number of DAPI staining cells.

### Cell cycle analyses of HaCaT keratinocytes

At 24 h after treatments, the HaCaT keratinocytes were harvested and washed with cold PBS and fixed with 80% ice-cold ethanol at −20 °C. After centrifugation, the cells were washed with ice-cold PBS twice and resuspended in PBS containing 0.2 mg/mL ribonuclease A and 20 μg/mL propidium iodide and incubated for 3 h at room temperature in the dark. The cell cycle distribution was analyzed by flow cytometry (EPICS®XL-MCL™; Beckman Coulter) using FlowJo software (Treestar, Inc., San Carlos, CA).

### Western blotting analyses

The total cellular proteins were extracted from treated keratinocytes with lysis buffer (1.5% SDS, 0.0625 M Tris–HCl and 1 mM Na_3_VO_4,_ pH 6.8) containing a protease inhibitor cocktail (Roche, Mannheim, Germany). For Western blotting analysis, 40 μg of extracted total cellular protein was subjected to 10% sodium dodecyl sulphate polyacrylamide gel electrophoresis. After blocking and washing, the membrane was incubated with antibodies including phosphorylated extracellular signal-regulated kinases (pERK), total-ERK (tERK), and GAPDH (Cell Signaling, Beverly, MA), followed by incubation with horseradish peroxidase (HRP)-labelled secondary antibody (1:5000; Jackson Immuno Research, West Baltimore Pike, PA) and developed using a commercial HRP substrate (Immobilon™ Western Chemiluminescent HRP Substrate; Millipore Corporation, Billerica, MA). The membranes were detected by ChemiDoc™ XRS (Bio-Rad Laboratories Inc., Hercules, CA).

### Statistics

For each experiment, at least three independent experiments were performed. SPSS for Windows (v19.0; SPSS Inc., Chicago, IL, USA) was used for statistical analysis, and results were expressed as mean ± SD. *Student t-test* was used for statistical evaluation between control and experimental groups, and P < 0.05 was considered statistically significant.

## Additional Information

**How to cite this article**: Lan, C.-C. E. *et al*. Irradiance-dependent UVB Photocarcinogenesis. *Sci. Rep.* **6**, 37403; doi: 10.1038/srep37403 (2016).

**Publisher’s note:** Springer Nature remains neutral with regard to jurisdictional claims in published maps and institutional affiliations.

## Supplementary Material

Supplementary Information

## Figures and Tables

**Table 1:** Cell cycle distribution of HaCaT keratinocytes 24 h after indicated UVB irradiation.

	G0/G1	S	G2/M
Control	40.8 ± 1.91	35.5 ± 2.19	23.7 ± 0.21
HI^1^ UVB	33.7 ± 1.48	41.4 ± 2.19	24.9 ± 0.14
LI^2^ UVB	39.1 ± 0.28	48.4 ± 0.57	12.5 ± 1.13

^1^HI: High irradiance.

^2^LI: Low irradiance.

Presented as (mean ± SD) from 3 independent experiments.

**Table 2:** Cell cycle distribution of ERK inhibitor (U0126) pretreated HaCaT keratinocytes 24 h after indicated UVB irradiation.

	G0/G1	S	G2/M
Control	64.7 ± 0.65	16.6 ± 0.81	18.7 ± 0.20
HI^1^ UVB	32.7 ± 0.89	42.2 ± 0.64	25.1 ± 0.79
LI^2^ UVB	33.9 ± 1.01	40.7 ± 0.92	25.4 ± 1.13

^1^HI: High irradiance.

^2^LI: Low irradiance.

Presented as (mean ± SD) from 3 independent experiments.
