# Supplementary material for: Irradiance-dependent UVB Photocarcinogenesis
Source: Sci Rep. 2016 Nov 21;6:37403. doi: 10.1038/srep37403 (PMC5116611; doi:10.1038/srep37403)

Irradiance-dependent UVB Photocarcinogenesis

Cheng-Che E. Lan[1-4], Ching-Shuang Wu[3-5], Shu-Mei Huang[1], Chin-Han Wu[1], Hsiao-Chi Lai[1], Yu-Ting Peng[1], Pao-Sheng Hou[1,3], Hui-Jun Yang[1], Gwo-Shing Chen[1]

1. Department of Dermatology, Kaohsiung Medical University Hospital, Department of Dermatology, College of Medicine, Kaohsiung Medical University, Kaohsiung, Taiwan
2. Department of Dermatology, Kaohsiung Municipal Ta-Tung Hospital, Kaohsiung Medical University, Kaohsiung, Taiwan
3. Lipid Science and Aging Research Center, Kaohsiung Medical University, Kaohsiung, Taiwan
4. Center of Environmental Medicine, Kaohsiung Medical University
5. Department of Medical Laboratory Science and Biotechnology, Kaohsiung Medical University, Kaohsiung, Taiwan

Corresponding author: Professor Cheng-Che E. Lan, e-mail: laneric@cc.kmu.edu.tw; Professor Gwo-Shing Chen, e-mail: d700086@cc.kmu.edu.tw

100 Shih-Chuan 1st Rd, Kaohsiung, Taiwan; Department of Dermatology, Kaohsiung Medical University Hospital, Kaohsiung Medical University

Tel: +8867 3121101 ext 6108

Supplementary file

Fig. S1. The emission spectrum of the irradiation source MEL308 as provided by the manufacturer.

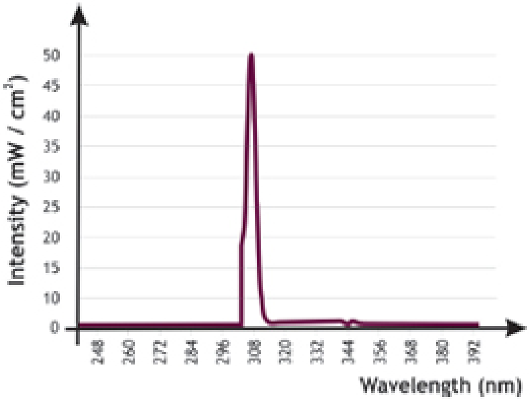

Fig. S2. The spectral irradiance of the UVB emitting source with and without neutral density filter was measured using a spectroradiometer (i-Spec™; B&W Tek, Inc., Newark, DE, USA). a.u. indicates arbitrary unit.

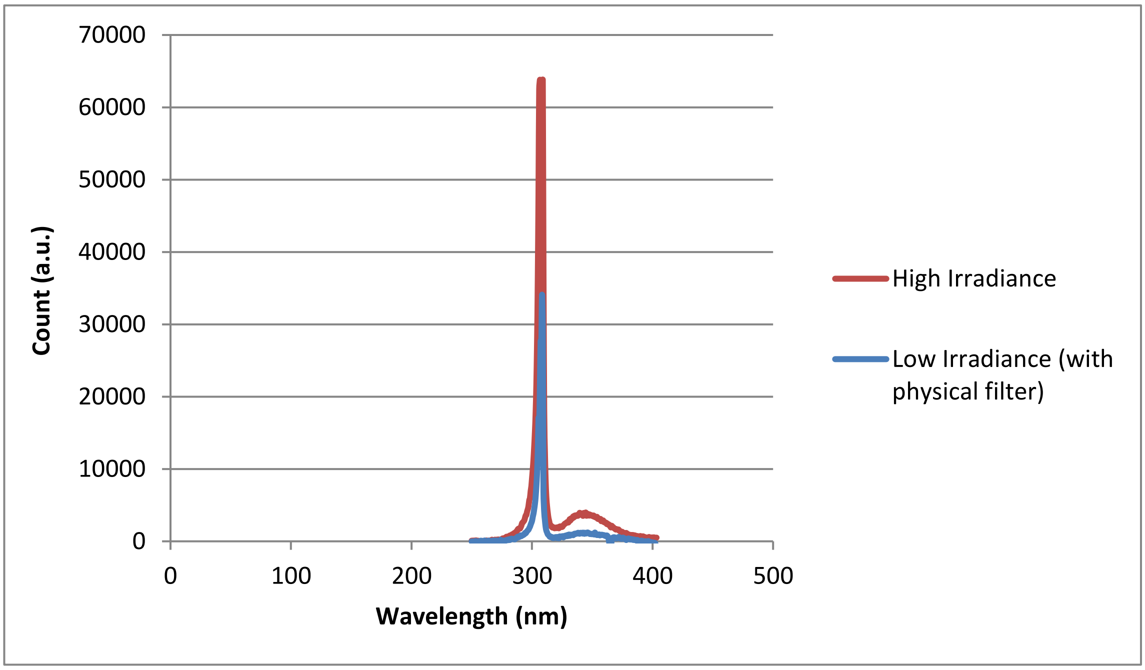

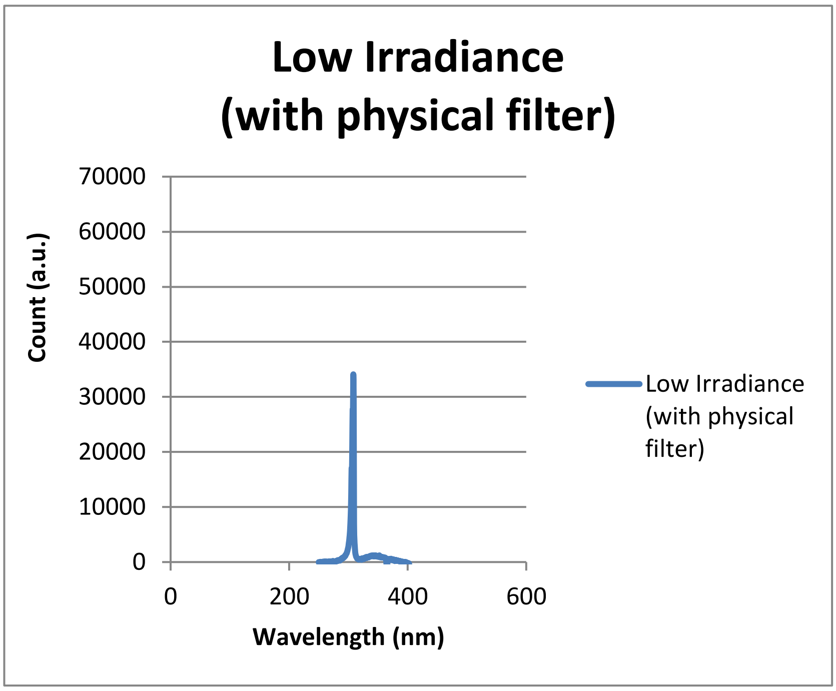
Low Irradiance
(with physical filter)
70000
60000
50000
40000
30000
20000
10000
0
0
200
400
600
Count (a.u.)
Wavelength (nm)
Low Irradiance (with physical filter)

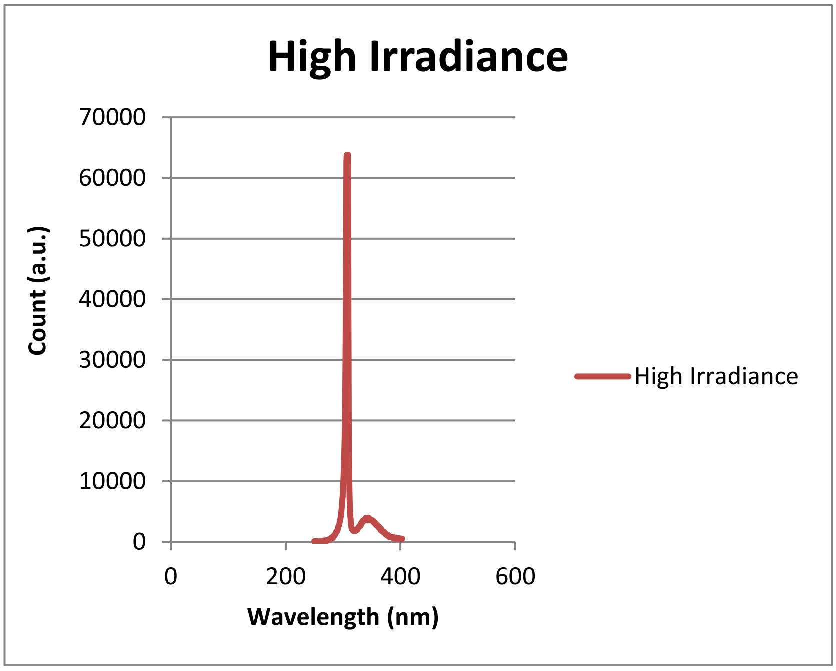
High Irradiance
70000
60000
50000
40000
30000
20000
10000
0
0
200
400
600
Count (a.u.)
Wavelength (nm)
High Irradiance

Fig S3. The expression of cyclopyrimidine dimer (CPD) of mice skin after indicated treatment. As demonstrated below (upper panel), the low irradiance (LI) UVB treated mice epidermis demonstrated higher portion of epidermal keratinocytes containing CPD at the time of biopsy as compared to their high irradiance (HI) treated counterpart. The quantitative analysis is shown in the lower panel. However, the difference between the LI and HI UVB treated groups did not achieve statistical significance (p=0.056)

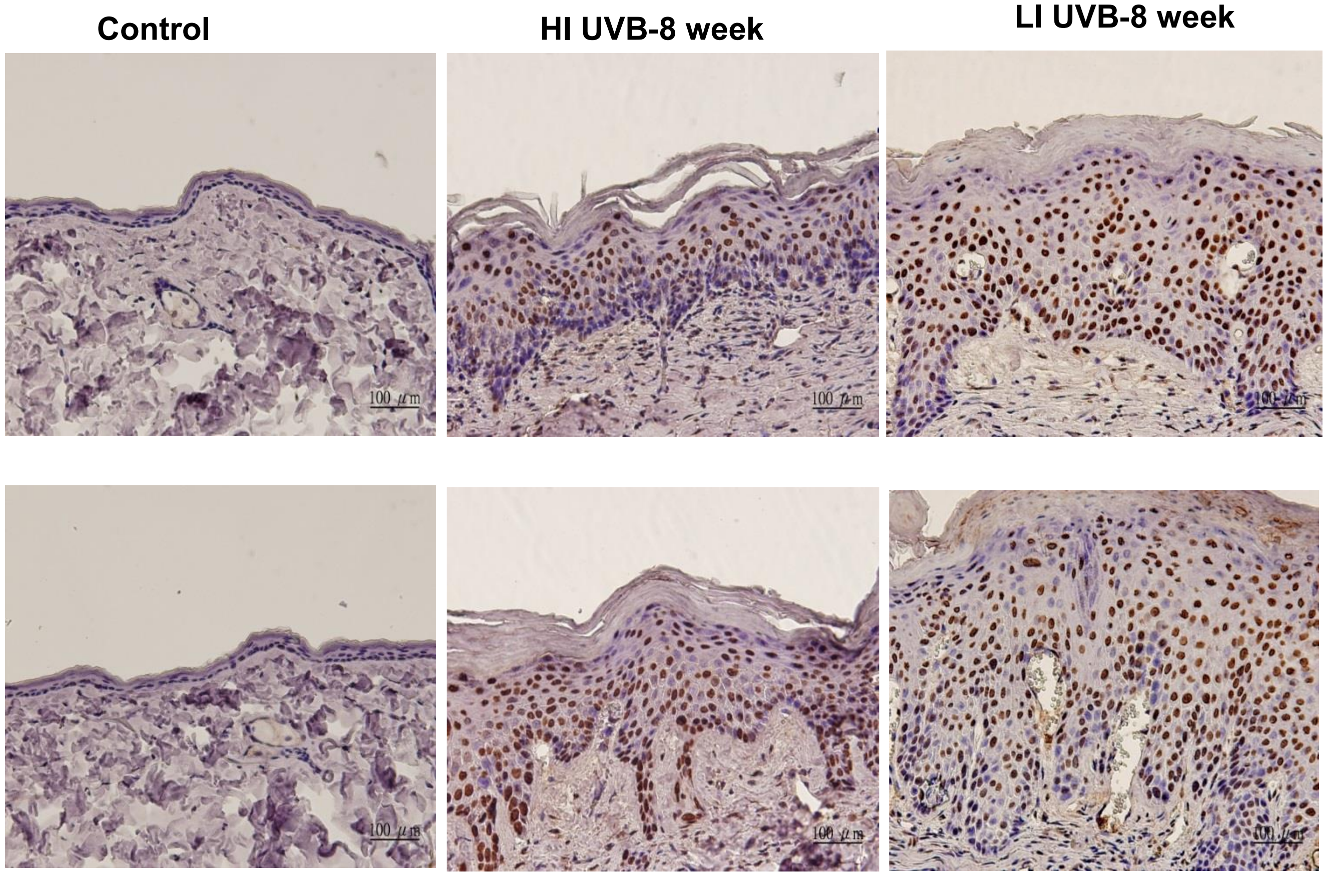

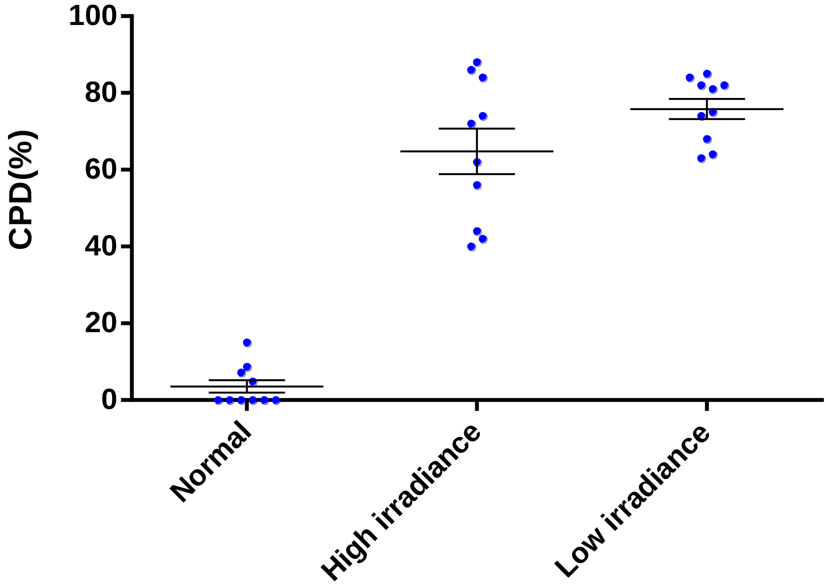

Supplement: Supplementary Information [file srep37403-s1.pdf]
